# Berry Consumption in Relation to Allostatic Load in US Adults: The National Health and Nutrition Examination Survey, 2003–2010

**DOI:** 10.3390/nu16030403

**Published:** 2024-01-30

**Authors:** Li Zhang, Joshua E. Muscat, Vernon M. Chinchilli, Penny M. Kris-Etherton, Laila Al-Shaar, John P. Richie

**Affiliations:** 1 Department of Public Health Sciences, Penn State Cancer Institute, Penn State College of Medicine, Pennsylvania State University, Hershey, PA 17033, USA; vchinchilli@pennstatehealth.psu.edu (V.M.C.); lshaar83@gmail.com (L.A.-S.); jrichie@pennstatehealth.psu.edu (J.P.R.); 2 Department of Nutritional Sciences, Pennsylvania State University, University Park, PA 16802, USA; pmk3@psu.edu

**Keywords:** berry consumption, allostatic load, biomarkers, stress, physiological dysregulations, antioxidant, polyphenol, NHANES, adults

## Abstract

Introduction: Berries are a rich source of antioxidant polyphenols and other nutrients that are associated with good health. Allostatic load (AL) is an aggregate measure of chronic stress-induced physiological dysregulations across cardiovascular, metabolic, autonomic, and immune systems; the extent of these dysregulations, collectively or in each system, can be characterized by a composite score or a domain score assessed by integrated biomarkers. It was hypothesized that the anti-inflammatory and other effects of berries lower AL. The association was determined between berry consumption and AL composite and domain scores in the 2003–2010 National Health and Nutrition Examination Survey (NHANES). Methods: Berry intake was measured using two 24 h dietary recalls collected from US adults in the 2003–2010 NHANES (*n* = 7684). The association with AL and its specific domains was examined using population weight-adjusted multivariable linear regression. Results: The mean AL composite scores for consumers of any berries (11.9), strawberries (11.6), and blueberries (11.6), respectively, were significantly lower than nonconsumers (12.3), after fully adjusting for sociodemographic, lifestyle, and dietary confounders. A significant dose-response relationship was determined between greater consumption of total berries, strawberries, and blueberries and lower mean AL composite scores (*p*-trend < 0.05, for all). Consistently, mean cardiovascular and metabolic domain scores remained significantly lower in the consumers of total berries (mean cardiovascular domain score: 4.73 versus 4.97 for nonconsumers; mean metabolic domain score: 2.97 versus 3.1), strawberries (4.73 versus 4.95; 2.99 versus 3.1), and blueberries (4.6 versus 4.95; 2.92 versus 3.11). Berry consumers also had significantly lower mean AL immune scores (1.52 versus 1.56) and lower mean AL autonomic scores (2.49 versus 2.57) than nonconsumers (initial sample: *n* = 15,620). Conclusions: The current study indicates that consumption of berries lowers the AL composite scores and potentially reduces stress-related disease risks in the US adult population.

## 1. Introduction

Individuals are susceptible to a variety of stressors, including environmental and psychosocial stressors from ordinary events, significant life challenges, and unhealthy lifestyle behaviors (e.g., poor sleep, physical inactivity, smoking, alcohol consumption, etc.). To adapt to situations perceived and interpreted as stressful and challenging, adaptive stress responses are prompted in the body to restore homeostasis [1,2]. However, chronic stress may induce excess secretion of primary stress hormones (e.g., catecholamines and glucocorticoids) that can be damaging, resulting in dysregulations in multiple physiological systems that culminate in disease outcomes over time [3,4]. Allostatic load (AL) is a multisystem measurement framework reflecting the incremental effects of stress on physiological risk. Measurement of AL is commonly conducted using biomarkers of multiple physiological systems affected by stress [5,6,7], including the dysregulation of cardiovascular, autonomic, metabolic, and immune systems [1,6,8,9]. AL can be characterized by a composite or domain score to reflect the severity of physiological dysregulation collectively or in specific systems [10,11,12].

Higher AL scores have consistently been related to a greater risk of impaired health and debilitating stress-related health conditions, such as physical and cognitive decline, obesity, diabetes, cardiovascular diseases, cancer, and mental disorders (e.g., depression, mood disorders, and anxiety) [13,14,15,16]. Higher mortality risk has been reported in adults who have higher AL scores [14,17,18,19,20,21].

A mounting interest has been displayed in identifying foods and their effects in mitigating chronic stress responses and stress-induced diseases. According to emerging findings from animal models and human trials, polyphenols (organic compounds ubiquitously found in plants) and polyphenol-rich foods, such as tea and chocolate, show promise for reducing stress responses and pro-inflammatory factors, as well as improving stress-related health conditions [22,23,24,25,26,27,28,29]. Berries are an excellent source of polyphenols and other nutrients that have potent antioxidant properties (e.g., vitamin C, vitamin E, and beta-carotene) and protect against inflammation and cardiometabolic disease [30,31,32]. The levels of specific polyphenols and other nutrients vary among berry types. Raspberries and blackberries, for example, have the highest levels of polyphenol ellagic acid, whereas strawberries have high levels of anthocyanins. Blueberry extracts alleviate stress responses in stressed laboratory rats, and blueberry drinks have been associated with improved mood in young adults and children [33,34].

The effects of berry consumption on AL in a typical diet have not been investigated. The hypothesis was that greater total berry consumption is associated with lower AL. Using a large, nationally representative sample of US adults from the NHANES, this cross-sectional research over a span of eight years could determine the association between berry consumption and physiological dysregulation from stress responses measured by AL scores.

## 2. Methods

### 2.1. Study Design

This analysis was performed using the combined four cycles (2003–2004, 2005–2006, 2007–2008, 2009–2010) of publicly available data from the NHANES. The NHANES, administered by the National Center for Health Statistics (NCHS), was a periodic survey initiated in 1960 and became a continuous program in 1999 [35]. The NHANES recruits a representative sample of the noninstitutionalized US population with a complex, multistage, probability sampling design [36] and collects demographic, dietary, and medical information through an at-home interview, a mobile examination center (MEC) visit, or a phone interview [37]. The MEC visit includes dietary interviews, complete medical examinations, and laboratory analysis of blood, urine and other tissue samples collected. Trained staff conduct in-person interviews and phone interviews (in the following 3–10 days with response rate: 75–80%) to collect one or two 24 h food recalls from the respondents, using the accuracy-enhanced automated multiple-pass method recommended by the USDA [36,38,39]. The Research Ethics Review Board of the NCHS has approved the NHANES protocol, and all respondents provided written consent. The present study was exempt from the Institutional Review Board approval.

### 2.2. Analytic Sample

The analysis initially included 15,620 American adults (20 years or older) who completed two 24 h recalls, excluding the pregnant women (*n* = 506), lactating women (*n* = 135), and participants who had improbable energy intake (*n* = 552; <800 or >4200 kcal for males and <500 or >3500 kcal for females) or missing values on at least one of the AL immune and autonomic biomarkers (*n* = 1379). Due to the analyses of AL composite scores along with cardiovascular and metabolic domain scores requiring assessments of the fasting biomarkers that were available from the individuals who only attended a morning examination, the final analyzed sample was restricted to the ones who attended the morning examination and provided complete information on the biomarkers, including examined fasting biomarkers (*n* = 7684) (Figure S1).

### 2.3. Berry Consumption and Berry Consumers

An algorithm to identify berry intake from the food records was developed and published previously by our research team since berries are often consumed in mixed and processed foods [40,41]. A manual search of the Food and Nutrient Database for Dietary Studies (FNDDS) food code description was conducted for food items that contained berries and berry subtypes (strawberries, blueberries, cranberries, raspberries, and blackberries) as part of a food group [42,43]. Berry-flavored alcoholic beverages were removed from the analysis.

The quantification of intakes of berries (berry subtypes) was converted from grams to cup-equivalents using cycle-specific releases of the USDA’s MyPyramid Equivalents Database (MPED 2.0) and the Food Patterns Equivalent Database (FPED) to be in line with the USDA 2015–2020 dietary guidelines for Americans [44,45,46]. Specifically, the conversion can be performed using the category “Citrus, melons, and berries” in MPED and FPED.

Berry consumers were the participants who reported intakes of berry (or berry subtype) fruits (>0 cup-equivalents) in at least one food recall.

### 2.4. Allostatic Load Score Composition

The AL score is an aggregated measure of 14 biomarkers across four multiple physiological systems (e.g., domains) to represent the severity of physiological dysregulation in response to chronic stressors. The components of the AL score encompassed the cardiovascular domain (low-density lipoprotein cholesterol: fasting LDL-cholesterol, high-density lipoprotein cholesterol: HDL-cholesterol, total cholesterol: TC, fasting glucose, fasting insulin, glycated hemoglobin: HbA1c); the autonomic domain (pulse rate, systolic blood pressure: SBP, diastolic blood pressure: DBP); the metabolic domain (insulin resistance: HOMA-IR, fasting triglycerides, waist circumference), and the immune domain (C-reactive protein: CRP, white blood cell counts: WBC) (Table S1). The selection of these biomarkers was based on previous research [10,11,12,14,21,47,48,49,50]. A 3-level score indicator (0: normal, 1: moderate, or 2: high) was assigned to each biomarker within each domain for each participant by either a clinically or empirically meaningful cut point or reliable evidence in the literature to indicate a threshold of disease risk [51,52,53,54,55,56,57,58,59,60,61,62,63,64,65,66,67,68,69,70,71,72,73,74,75]. The risk indicator for each biomarker was then summed in each domain to form AL domain scores and finally aggregated for each respondent to create an AL composite score (range: 0–28). A higher AL score indicates a higher extent of dysregulation [10,14].

All adult respondents were eligible for physical examinations and most clinical exam measurements; however, fasting laboratory measurements such as LDL-cholesterol, triglycerides, glucose, and insulin were collected only from the respondents who attended a morning examination. Specimen collection and laboratory procedures are documented in NHANES Laboratory/Medical Technologists Procedures Manual [76]. HOMA-IR was calculated with the formula: fasting insulin (mU/L) × fasting glucose (mmol/L)/22.5.

### 2.5. Covariates

We selected the confounders a priori that would be associated with stress or AL based on previous research [13,14,47,77,78,79]. Self-reported sociodemographic factors were defined as follows: age (years), sex, five-level race/ethnicity, four-level educational attainment, and three-level poverty-to-income ratio (PIR). Lifestyle factors were also self-reported, including current smoking status (Yes/No), physical activity (sedentary, low: below minimum recommendations, moderate: 150–300 min of moderate-intensity or 75–150 min of vigorous activity as recommended, or high: above the moderate levels), marriage status (married or living together or not), use of glucose-lowering, lipid-lowering, or blood pressure-lowering medication (Yes/No), and body mass index (BMI, low or normal: <25, overweight: 25–30, or obesity: ≥30 kg/m^2^). To avoid multicollinearity, we modified the healthy eating index (HEI-2015, a summary index for assessing dietary patterns) by removing berries. Dietary factors were treated as continuous measurements in the analysis, including alcohol use, total energy intake, and modified HEI-2015.

### 2.6. Statistical Analysis

All the analyses were performed using SAS (version 9.4) and a 2-tailed α-level of 0.05. False discovery rate adjusted *p* values were reported when adjusting for multiple comparisons. To produce the nationally representative estimates, all statistical analyses were performed employing survey procedures with appropriate survey weights, strata, and primary sampling units to account for the complex survey design of NHANES [80]. The comparison by berry consumer status was based on participants who provided at least one dietary recall. Therefore, the day 2 dietary weights were adjusted for complex study design and nonresponse, and the outcomes involving fasting laboratory biomarkers were analyzed using fasting subsample weights, as recommended by the analytical guidelines of NHANES [81]. For missing values on sociodemographic and lifestyle covariates (missing rate: 19.7%), a single imputation with the hot-deck technique was performed using PROC SURVEYIMPUTE.

The Rao-Scott χ^2^ test was adopted to compare the categorical demographic and lifestyle characteristics based on consumer status. Multivariable-adjusted linear regression models were built to estimate least square means (LSM) and 95% confidence intervals (CIs) for AL composite score and AL domain scores between berry (and subtype) consumers and nonconsumers, and across the category of berry consumption (0 cup-equivalent, ≤50th percentile of berry intake, and >50th percentile) using the SAS procedure PROC SURVEYREG. To further assess the association between the berry intake and each biomarker relevant to AL, the same models were used to estimate the effect of berry consumption on the mean level of each biomarker. The models were adjusted for these potential confounders: age (years), categorical sociodemographic factors (sex, race/ethnicity, marital status, educational attainment, and PIR), categorical lifestyle factors (current smoking status, physical activity level, BMI, and the use of medications to lower glucose, lipids, or blood pressure: Y/N), and continuous dietary factors (total energy intake, alcohol intake, and modified HEI-2015). Total sugar intake (g/day) was also adjusted for when assessing total berry consumption and AL composite scores for its significant confounding effect. *p* values for the t-test were used to compare consumers versus nonconsumers with regression adjustment for covariates. A test for a linear trend was performed by incorporating the category of berry consumption as a continuous variable in the model. The association was examined between berry consumption and each individual AL biomarker.

## 3. Results

### 3.1. Population Characteristics

Among the eligible 7684 respondents, approximately 19.3% of adults (*n* = 1485, 61.9%: female) consumed berries (>0 cup-equivalents) on either or both of the two 24 h recalls (Table 1). Most berry consumers were women, non-Hispanic whites, smokers, married, wealthier, well-educated, and physically active, and they were less likely to be classified as obese. Berry consumers also reported higher mean age, diet quality, and energy intake, as well as lower mean alcohol intake than nonconsumers.

### 3.2. AL Composite Scores Associated with Berry Consumption

Table 2 presents the mean AL composite scores between berry (including subtype) consumers and nonconsumers. Compared to nonconsumers, consumers of total berries (mean AL composite score = 11.85, mean difference = −0.46, 95% CI: −0.76~−0.17; *p* = 0.0026), strawberries (mean = 11.59, mean difference = −0.36, 95% CI: −0.64~−0.08; *p* = 0.013), and blueberries (mean = 11.64, mean difference = −0.62, 95% CI: −1.07~−0.17; *p* = 0.008), respectively, had significantly lower mean AL composite scores, after fully adjusting for sociodemographic, lifestyle, dietary and health confounders. Relative to categorized berry intake, a significant dose–response relationship was also observed between greater consumption of total berries (*p* = 0.0007), strawberries (*p* = 0.02), and blueberries (*p* = 0.001) and lower mean AL composite scores (Table 3). To further understand how socioeconomic status affects AL between consumers and nonconsumers, we conducted a stratified analysis of the three levels of poverty-to-income ratio and four levels of education. We found that AL level did not differ significantly by PIR and education level between berry consumers and nonconsumers.

### 3.3. AL Domain Scores: Berry Consumers vs. Nonconsumers

The mean difference in AL cardiovascular and metabolic domain scores by berry consumer status is shown in Table 4. Consistent with the results of the mean AL composite scores, the mean cardiovascular domain score and mean metabolic domain score remained significantly lower in the consumers of total berries (mean cardiovascular domain score: 4.73 for berry consumers versus 4.97 for nonconsumers; mean metabolic domain score: 2.97 versus 3.1), strawberries (4.73 versus 4.95; 2.99 versus 3.1), and blueberries (4.6 versus 4.95; 2.92 versus 3.11) in the fully adjusted model 2.

The comparisons of the mean AL immune and autonomic domain scores between berry consumers and nonconsumers are presented in Table 5 (based on a final sample of 7684). Because the assessment of the individual AL immune and autonomic domains does not require fasting biomarkers like the composite AL score, we were able to use the larger initial sample of 15,620 for this analysis (Table S2). Among 15,620 respondents, berry consumers had a significantly lower mean AL immune score (1.52 versus 1.56; mean difference = −0.05, *p* = 0.029) and a lower mean AL autonomic score (2.49 versus 2.57; mean difference = −0.07, *p* = 0.026) than nonconsumers in the fully adjusted model. A significantly lower mean autonomic score was also observed for blackberry consumers versus nonconsumers (2.34 versus 2.63; mean difference = −0.29, *p* = 0.007). However, no significant differences in the mean AL immune or autonomic domain scores were observed between consumers of total berries (including berry subtypes) and nonconsumers for the final sample, possibly due to the restricted sample size (Table 5).

### 3.4. Individual AL Biomarker Analysis

Consistent with the findings regarding AL composite and domain scores, berry consumers had significantly lower mean levels of biomarkers in each AL domain in the full model: cardiovascular domain (LDL-cholesterol, fasting glucose, fasting insulin), metabolic domain (triglycerides, HOMA-IR, waist circumference), immune domain (WBC), and autonomic domain (pulse rate) (Table S3).

In terms of subtype berries, consumers of strawberries and blueberries, respectively, had significantly lower mean levels of fasting glucose and waist circumference than nonconsumers. Significantly lower mean levels of fasting insulin, HOMA-IR, and triglycerides were also observed for blueberry consumers compared with nonconsumers. Additionally, mean fasting glucose level was also lower in raspberry consumers versus nonconsumers.

Compared to nonconsumers, blackberry consumers had lower mean levels of triglycerides and CRP, while the mean WBC was significantly lower in cranberry juice consumers. Furthermore, mean pulse rate was significantly lower in consumers of blueberries, cranberries, blackberries, and cranberry juice consumers, respectively (*p* < 0.05, for all).

## 4. Discussion

This study found that total berry consumption was associated with lower AL. When examining specific AL domains, the effects were observed primarily for cardiovascular and metabolic systems. In addition, berry type was associated with AL. Greater intakes of blueberries and strawberries (main source of anthocyanidin intake) were significantly related to a lower AL composite score, and lower cardiovascular and metabolic domain scores. The findings agree with the emerging evidence that polyphenol-rich foods can reduce stress [22].

There have been few human studies on berry consumption and stress, whereas feeding studies have examined associations between berry consumption and individual cardiometabolic risk factors. Meta-analyses of randomized clinical trials demonstrated that berry supplementation reduces cardiovascular and metabolic disease risk markers (e.g., total cholesterol, triglycerides, LDL-cholesterol, fasting glucose, fasting insulin, triglycerides, and HbA1c) [82,83]. These findings have been attributed to the flavonoids (especially anthocyanins) in berries that are potent antioxidants and their effects on improving insulin sensitivity and glycemic and lipid profiles [84,85,86]. Another meta-analysis of 32 RCTs of both berries and other foods that contain high levels of anthocyanins also reported reduced levels of lipids and blood pressure [87]. There may be other components in berries that have a beneficial effect on cardiometabolic factors. Dietary fibers, which are abundant in whole berries, decrease glucose absorption [88].

The present findings demonstrate that berries, when included in an average American diet (not solely supplemented by berries or berry products), can also play a beneficial role in mitigating dysregulations in physiological systems as well as preventing cardiovascular and metabolic alterations and/or disorders. Specifically, the significant associations between greater intakes of strawberries and blueberries and decreased AL scores mirrored the associations found for total berry consumption. These results are suggestive of the protective effects of anthocyanins against multisystemic dysregulations, especially in cardiovascular and metabolic systems.

The significantly lower mean immune and autonomic domain scores, respectively, were detected in berry consumers compared to nonconsumers (*n* = 15,620). Similar to our findings, previous studies have reported that berry consumption was related to reduced levels of blood pressure (SBP and DBP) and CRP, the indicators of improved autonomic and immune functions [82,89,90,91]. Our research further suggests that the significantly decreased levels of pulse rate and WBC in berry consumers contribute to favorable autonomic and immune functions compared to nonconsumers. Since pulse rate and WBC have not been explored in prior berry research, these findings need to be validated in future clinical trials.

Reliable biological evidence supports our finding that consumption of polyphenol-rich berries may be related to lower AL scores, which reflect the extent of multisystemic dysregulations. Berries are rich in bioactive compounds, which likely alleviate stress responses through reduced neuroinflammation [92,93] and modulate brain-derived neurotrophic factors and the hypothalamic-pituitary-adrenal axis in the hippocampus [23,94,95]. Dietary polyphenols have also been found to interact with gut microbiota through the gut–brain–axis signaling pathway. This modulation fosters resilience to stress-induced physiological changes, reducing the severity of stress-induced dysregulation and AL scores [95,96].

### Study Strengths and Limitations

The strengths of this study include the use of a large nationally representative sample of US adults that used two 24-h recalls assessing dietary intake. A composite and a battery of AL biomarkers were used, reflecting multiple physiological systems that contribute to stress [7,10,48]. The use of multiple biomarkers takes into consideration the interconnective contribution of physiological systems important in stress biology relating to disease pathogenesis and overall health [5,8,13]. Integrating autonomic, immune/inflammatory, metabolic, and cardiovascular biomarkers for constructing validated summative AL scores can characterize the extent of stress-induced, cumulative alterations across physiological systems, allowing for quantifying multisystemic physiological dysregulations [8,14,48]. AL reflecting these dysregulations is a comprehensive multisystemic approach that may enhance the accuracy of the risk assessment of diseases and facilitate the understanding of stress-related health conditions/diseases and comorbidities [13,14,97,98], which is limited in individual biomarker studies. The AL score has been proven to better predict mortality and functional decline than individual biomarkers or a cluster-like metabolic syndrome (presence of at least three out of five metabolic biomarkers) [5,14,99,100,101]. Further, a cumulative composite AL score is more statistically sensitive and less prone to errors than the commonly used dichotomous approach for assessing individual biomarkers [102].

The present study has limitations. First, allostatic load scores were determined based on individual physiological biomarkers measured at one point in time in NHANES, which may not account for time-varying changes in each biomarker or rule out the possibility for biomarker risk misclassification. The study was also potentially confined by the availability of these biomarkers (especially fasting biomarkers) and missing dietary information collected in NHANES. Further, the self-reported dietary information from 24 h recalls may contain some random misclassification and misestimation errors about berry intake, which could bias our results. Finally, residual confounding cannot be excluded, though many potential confounders have been adjusted in the model. Future studies, therefore, should evaluate different populations using other diet assessment methods for individual berries and total berry consumption to replicate and validate our results with similar AL indexes.

## 5. Conclusions

In conclusion, using multiple biomarkers of stress-induced physiological dysregulation to produce a composite AL score, higher consumption of berries was associated with reduced AL in American adults. Increasing berry intake is a simple dietary modification that could reduce stress-related morbidity/comorbidity and promote health.

## Figures and Tables

**Table 1:** Sociodemographic and lifestyle characteristics of berry consumers versus nonconsumers in NHANES (2003–2010), *n* = 7684.

Characteristics	Consumers (*n* = 1485)	Nonconsumers (*n* = 6199)	*p* Value
Sex (Female), %	61.9 (59.0, 64.7)	49.5 (48.1, 51.0)	<0.0001
Race/ethnicity, %			<0.0001
Non-Hispanic White	83.4 (80.2, 86.7)	69.4 (65.8, 73.0)	
Non-Hispanic Black	5.5 (4.0, 6.9)	11.7 (10.0, 13.5)	
Mexican American	4.6 (3.3, 5.9)	8.6 (6.7, 10.4)	
Other Hispanic	3.1 (1.9,4.2)	4.4 (3.1, 5.7)	
Other	3.4 (1.9, 5.0)	5.9 (4.9, 6.9)	
BMI, %			<0.0001
<25	35.5 (32.9, 38.0)	30.7 (29.0,32.3)	
25–30	36.1 (33.5. 38.7)	33.0 (31.3.34.8)	
≥30	28.4 (26.2, 30.7)	36.3 (34.6, 38.0)	
PIR, %			<0.0001
<1.3	11.4 (9.7, 13.1)	20.5 (18.8, 22.3)	
1.3–1.85	33.0 (29.6, 36.3)	39.5 (37.1, 41.8)	
>1.85	55.6 (52.3, 58.9)	40.0 (37.5, 42.6)	
Education, %			<0.0001
Less than High School	8.9 (6.9, 10.8)	19.3 (17.8, 20.9)	
High school	20.3 (17.4, 23.3)	26.2 (24.3, 28.0)	
Some college	29.7 (26.4, 33.0)	30.6 (28.8, 32.3)	
≥4-year degree	41.1 (36.9, 45.4)	23.9 (21.6, 26.2)	
Married, or w/a partner (Yes), %	69.7 (66.6, 72.8)	64.7 (62.9, 66.6)	0.002
Physical activity, %			0.0002
Sedentary	13.4 (11.1, 15.6)	18.5 (16.8, 20.2)	
Low	19.8 (17.3, 22.2)	21.7 (20.3, 23.2)	
Moderate	19.0 (16.6, 21.4)	15.9 (14.7, 17.1)	
High	47.8 (45.0, 50.7)	43.9 (42.0, 45.7)	
Current smoker (Yes), %	13.1 (11.0, 15.2)	23.4 (21.6, 25.3)	<0.0001
Lipid medication (Yes), %	21.8 (19.2, 24.4)	22.2 (21.2, 23.3)	0.749
Blood pressure medication (Yes), %	25.8 (22.7, 28.8)	25.7 (23.9, 27.6)	0.966
Glucose medication (Yes), %	3.6 (2.4, 4.9)	3.6 (3.1, 4.3)	0.981
Age, mean ± S.E., y	50.3 ± 0.6	46.9 ± 0.4	<0.0001
Energy, kcal	2073.4 ± 24.0	2068.8 ± 15.3	<0.0001
Alcohol, g	8.3 ± 0.7	8.9 ± 0.5	<0.0001
HEI-2015	57.9 ± 0.5	51.6 ± 0.3	<0.0001

**Table 2:** The least square means (LSM) of allostatic load composite scores for total and individual berry consumers versus nonconsumers, *n* = 7684.

Total and Subtype Berries	Consumers LSM (95% CI)	Nonconsumers LSM (95% CI)	Mean Difference (95% CI)	*p*-Value
Berries				
Model 1	9.42 (9.14, 9.7)	10.66 (10.49, 10.84)	−1.24 (−1.54, −0.94)	<0.0001
Model 2	11.85 (11.41, 12.29)	12.31 (11.96, 12.66)	−0.46 (−0.76, −0.17)	0.0026
Strawberries				
Model 1	9.54 (9.16, 9.92)	10.56 (10.38, 10.74)	−1.02 (−1.44, −0.6)	<0.0001
Model 2	11.59 (11.18, 11.99)	11.94 (11.65, 12.24)	−0.36 (−0.64, −0.08)	0.013
Blueberries				
Model 1	9.22 (8.73, 9.70)	10.52 (10.35, 10.69)	−1.30 (−1.80, −0.80)	<0.0001
Model 2	11.64 (11.08, 12.21)	12.26 (11.91, 12.62)	−0.62 (−1.07, −0.17)	0.008
Cranberries				
Model 1	8.98 (8.25, 9.71)	10.48 (10.32, 10.64)	−1.50 (−2.20, −0.80)	<0.0001
Model 2	12.33 (11.58, 13.08)	12.21 (11.85, 12.56)	0.12 (−0.56, 0.80)	0.719
Raspberries				
Model 1	10.24 (9.26, 11.22)	10.45 (10.29, 10.61)	−0.21 (−1.17, 0.75)	0.663
Model 2	12.15 (11.41, 12.88)	11.99 (11.71, 12.26)	0.16 (−0.50, 0.82)	0.626
Blackberries				
Model 1	8.85 (7.51, 10.20)	10.46 (10.30, 10.62)	−1.60 (−2.94, −0.27)	0.019
Model 2	12.53 (11.62, 13.44)	12.16 (11.81, 12.51)	0.37 (−0.44, 1.18)	0.362
Cranberry juice				
Model 1	9.90 (9.32, 10.49)	10.49 (10.33, 10.65)	−0.58 (−1.14, −0.03)	0.04
Model 2	11.87 (11.28, 12.47)	12.23 (11.88, 12.58)	−0.35 (−0.86, 0.15)	0.169

Model 1 adjusted for age, sex, and race/ethnicity. Model 2 further adjusted for education, marriage status, family poverty-to-income ratio, physical activity, smoking status, alcohol consumption, total sugar intake (for berries only), total energy, modified healthy eating index, medication for lowering glucose and lipids, and BMI.

**Table 3:** Dose–response relationship between berry consumption (cup-equivalents) and mean AL composite score in US Adults, *n* = 7684.

Berry Type		Nonconsumption	Low LSM (95% CI)	High LSM (95% CI)	P*_trend_*
Berries	*n*	6199	754	731	
	Median (Range), cup-equivalents	0	0.06 (≤0.17)	0.35 (0.18, 3.95)	
	Mean ± S.E.	0	0.07 ± 0.05	0.45 ± 0.34	
	Model 1	10.71(10.54, 10.87)	9.46 (9.08, 9.84)	9.45 (9.08, 9.83)	<0.0001
	Model 2	12.1 (11.8, 12.4)	11.5 (11.2, 11.9)	11.6 (11.3, 12.0)	0.0007
Strawberries	*n*	6755	489	440	
	Median (Range), cup-equivalents	0	0.09 (≤0.19)	0.36 (0.2, 2.01)	
	Mean ± S.E.	0	0.09 ± 0.06	0.46 ± 0.29	
	Model 1	10.6 (10.4, 10.8)	9.6 (9.1, 10.0)	9.6 (9.0, 10.1)	0.0002
	Model 2	12.1 (11.8, 12.3)	11.6 (11.2, 12.0)	11.7 (11.4, 12.1)	0.02
Blueberries	*n*	7174	272	238	
	Median (Range), cup-equivalents	0	0.05 (≤0.11)	0.24 (0.11, 2.53)	
	Mean ± S.E.	0	0.05 ± 0.03	0.31 ± 0.28	
	Model 1	10.6 (10.4, 10.7)	9.6 (8.9, 10.3)	8.9 (8.2, 9.6)	<0.0001
	Model 2	12.1 (11.8, 12.3)	11.7 (11.1, 12.2)	11.2 (10.6, 11.8)	0.0011
Cranberries	*n*	7518	81	85	
	Median (Range), cup-equivalents	0	0.04 (≤0.11)	0.22 (0.12, 1.30)	
	Mean ± S.E.	0	0.04 ± 0.03	0.29 ± 0.21	
	Model 1	10.5 (10.4, 10.7)	9.2 (8.0, 10.4)	8.8 (8.0, 9.6)	<0.0001
	Model 2	12.0 (11.8, 12.3)	11.9 (11.0, 12.8)	11.8 (11.0, 12.5)	0.3787
Cranberry juice	*n*	7250	219	215	
	Median (Range), cup-equivalents	0	0.02 (≤0.05)	0.12 (0.05, 1.24)	
	Mean ± S.E.	0	0.02 ± 0.01	0.17 ± 0.17	
	Model 1	10.5 (10.4, 10.7)	9.8 (9.0, 10.5)	10.0 (9.2, 10.8)	0.113
	Model 2	12.0 (11.8, 12.3)	11.9 (11.2, 12.5)	11.6 (10.9, 12.2)	0.112
Raspberries	*n*	7580	56	48	
	Median (Range), cup-equivalents	0	0.09 (≤0.19)	0.32 (0.20, 2.58)	
	Mean ± S.E.	0	0.09 ± 0.06	0.38 ± 0.19	
	Model 1	10.5 (10.3, 10.9)	10.9 (9.6, 12.2)	9.8 (8.3, 11.3)	0.4353
	Model 2	12.0 (11.7, 12.3)	12.2 (11.1, 13.3)	12.1 (11.3, 12.9)	0.7672
Blackberries	*n*	7631	29	24	
	Median (Range), cup-equivalents	0	0.13 (≤0.24)	0.35 (0.25, 1.32)	
	Mean ± S.E.	0	0.12 ± 0.06	0.43 ± 0.23	
	Model 1	10.5 (10.4, 10.7)	8.6 (7.4, 9.7)	9.3 (7.0, 11.7)	0.1269
	Model 2	12.0 (11.8, 12.3)	12.0 (10.8, 13.1)	12.1 (11.1, 13.0)	0.9532

Berry intake categories: nonconsumption (intake = 0 cup-equivalents), low (intake ≤ 50th percentile), high (intake > 50th percentile). Model 1 adjusted for age, sex, and race/ethnicity. Model 2 further adjusted for education, marital status, family PIR, physical activity, smoking status, alcohol, total sugar (for total berries only), total energy, modified HEI, medication for lowering glucose and lipids, and BMI. LSM: least square means. CI: confidence interval.

**Table 4:** Comparison of the least square means of allostatic load (cardiovascular and metabolic) domain scores: total and individual berry consumers versus nonconsumers, *n* = 7684.

Total and Subtype Berries	Cardiovascular Domain (HDL-C, LDL-C, Glucose, Insulin, TC, HbA1c) (0–12)				Metabolic Domain (HOMAir, Triglycerides, Waist Circumference) (0–6)			
	Consumers LSM (95% CI)	Non-Consumers LSM (95% CI)	Difference Estimate (95% CI)	*p*	Consumers LSM (95% CI)	Non-Consumers LSM (95% CI)	Difference Estimate (95% CI)	*p*
Berries								
Model 1	3.91 (3.78, 4.05)	4.40 (4.32, 4.49)	−0.49 (−0.64, −0.34)	<0.0001	2.20 (2.09, 2.32)	2.62 (2.55, 2.68)	−0.41 (−0.53, −0.30)	<0.0001
Model 2	4.73 (4.52, 4.93)	4.97 (4.78, 5.17)	−0.25 (−0.38, −0.11)	0.0004	2.97 (2.83, 3.11)	3.13 (3.01, 3.24)	−0.15 (−0.26, −0.04)	0.0078
Strawberries								
Model 1	3.94 (3.76, 4.12)	4.37 (4.28, 4.46)	−0.43 (−0.63, −0.23)	<0.0001	2.22 (2.08, 2.36)	2.59 (2.52, 2.65)	−0.37 (−0.51, −0.23)	<0.0001
Model 2	4.73 (4.49, 4.98)	4.95 (4.76, 5.15)	−0.22 (−0.38, −0.06)	0.0092	2.99 (2.83, 3.15)	3.11 (2.99, 3.22)	−0.12 (−0.23, −0.01)	0.0354
Blueberries								
Model 1	3.80 (3.53, 4.06)	4.35 (4.27, 4.43)	−0.55 (−0.81, −0.29)	<0.0001	2.12 (1.93, 2.32)	2.57 (2.51, 2.64)	−0.45 (−0.65, −0.25)	<0.0001
Model 2	4.63 (4.34, 4.92)	4.95 (4.76, 5.14)	−0.32 (−0.55, −0.09)	0.008	2.92 (2.75, 3.10)	3.11 (2.99, 3.23)	−0.19 (−0.34, −0.03)	0.0233
Cranberries								
Model 1	3.98 (3.61, 4.35)	4.33 (4.24, 4.41)	−0.34 (−0.71, 0.02)	0.0648	2.03 (1.74, 2.32)	2.56 (2.49, 2.62)	−0.53 (−0.81, −0.25)	0.0003
Model 2	5.03 (4.64, 5.41)	4.94 (4.74, 5.13)	0.09 (−0.27, 0.45)	0.613	2.94 (2.74, 3.14)	3.07 (2.98, 3.16)	−0.13 (−0.33, 0.07)	0.098
Raspberries								
Model 1	4.17 (3.69, 4.64)	4.28 (4.19, 4.37)	−0.12 (−0.59, 0.36)	0.629	2.45 (2.12, 2.78)	2.55 (2.48, 2.61)	−0.10 (−0.42, 0.23)	0.55
Model 2	5.07 (4.51, 5.61)	4.94 (4.74, 5.13)	0.13 (−0.36, 0.61)	0.605	3.04 (2.76, 3.31)	3.09 (2.98, 3.21)	−0.06 (−0.33, 0.22)	0.675
Blackberries								
Model 1	4.29 (3.62, 4.95)	4.32 (4.24, 4.40)	−0.03 (−0.69, 0.63)	0.918	1.90 (1.36, 2.43)	2.55 (2.49, 2.61)	−0.65 (−1.19, −0.12)	0.017
Model 2	5.43 (4.85, 6.02)	4.92 (4.73, 5.12)	0.51 (−0.06, 1.08)	0.078	2.98 (2.63, 3.32)	3.08 (2.98, 3.17)	−0.10 (−0.45, 0.24)	0.244
Cranberry juice								
Model 1	4.17 (3.90, 4.43)	4.33 (4.25, 4.41)	−0.16 (−0.41, 0.09)	0.195	2.30 (2.09, 2.50)	2.56 (2.50, 2.63)	−0.27 (−0.47, −0.07)	0.0102
Model 2	4.89 (4.60, 5.17)	4.94 (4.74, 5.14)	−0.05 (−0.28, 0.18)	0.652	2.95 (2.73, 3.17)	3.11 (2.99, 3.23)	−0.15 (−0.36, 0.05)	0.141

Model 1 adjusted for age, sex, and race/ethnicity. Model 2 further adjusted for education, marital status, family PIR, physical activity, smoking status, alcohol, total energy, modified HEI, medication for lowering glucose, lipids, and blood pressure, and BMI.

**Table 5:** Comparison of the least square means of allostatic load (immune and autonomic) domain scores: total and individual berry consumers versus nonconsumers, *n* = 7684.

Total and Subtype Berries	Immune Domain (White Blood Cells Counts, CRP) (0–4)				Autonomic Domain (Pulse Rate, Blood Pressure) (0–6)			
	Consumers LSM (95% CI)	Non-Consumers LSM (95% CI)	Difference Estimate (95% CI)	*p* Value	Consumers LSM (95% CI)	Non-Consumers LSM (95% CI)	Difference Estimate (95% CI)	*p* Value
Berries								
Model 1	1.28 (1.22, 1.35)	1.43 (1.39, 1.47)	−0.15 (−0.22, −0.08)	<0.0001	2.06 (1.97, 2.15)	2.25 (2.19, 2.32)	−0.20 (−0.28, −0.11)	<0.0001
Model 2	1.47 (1.39, 1.54)	1.51 (1.45, 1.57)	−0.04 (−0.10, 0.02)	0.166	2.48 (2.40, 2.57)	2.54 (2.46, 2.61)	−0.06 (−0.12, 0.01)	0.093
Strawberries								
Model 1	1.31 (1.24, 1.38)	1.42 (1.38, 1.45)	−0.11 (−0.18, −0.03)	0.005	2.11 (2.0, 2.23)	2.23 (2.17, 2.29)	−0.12 (−0.23, −0.01)	0.035
Model 2	1.48 (1.40, 1.56)	1.49 (1.44, 1.55)	−0.01 (−0.07, 0.06)	0.838	2.56 (2.45, 2.68)	2.59 (2.51, 2.67)	−0.03 (−0.14, 0.07)	0.506
Blueberries								
Model 1	1.28 (1.17, 1.39)	1.41 (1.38, 1.45)	−0.13 (−0.24, −0.02)	0.026	2.06 (1.91, 2.21)	2.23 (2.17, 2.29)	−0.17 (−0.33, −0.01)	0.036
Model 2	1.46 (1.35, 1.57)	1.50 (1.44, 1.55)	−0.04 (−0.14, 0.06)	0.429	2.50 (2.35, 2.66)	2.53 (2.46, 2.60)	−0.03 (−0.18, 0.12)	0.681
Cranberries								
Model 1	1.17 (1.01, 1.33)	1.41 (1.38, 1.44)	−0.24 (−0.39, −0.08)	0.003	1.81 (1.56, 2.06)	2.23 (2.17, 2.29)	−0.42 (−0.66, −0.18)	0.0008
Model 2	1.42 (1.27, 1.57)	1.50 (1.44, 1.55)	−0.08 (−0.21, 0.06)	0.276	2.36 (2.13, 2.58)	2.52 (2.45, 2.59)	−0.17 (−0.39, 0.06)	0.14
Raspberries								
Model 1	1.37 (1.14, 1.60)	1.38 (1.35, 1.42)	−0.01 (−0.24, 0.21)	0.898	2.18 (1.95, 2.41)	2.22 (2.16, 2.28)	−0.04 (−0.26, 0.18)	0.716
Model 2	1.58 (1.38, 1.79)	1.50 (1.44, 1.55)	0.09 (−0.12, 0.29)	0.403	2.47 (2.32, 2.62)	2.52 (2.45, 2.59)	−0.05 (−0.19, 0.09)	0.469
Blackberries								
Model 1	1.01 (0.73, 1.28)	1.41 (1.37, 1.44)	−0.40 (−0.67, −0.13)	0.004	1.77 (1.37, 2.16)	2.22 (2.17, 2.28)	−0.45 (−0.85, −0.06)	0.024
Model 2	1.31 (1.07, 1.54)	1.50 (1.44, 1.56)	−0.19 (−0.41, 0.03)	0.085	2.32 (2.04, 2.6)	2.53 (2.46, 2.6)	−0.21 (−0.48, 0.07)	0.146
Cranberry juice								
Model 1	1.34 (1.23, 1.44)	1.41 (1.38, 1.45)	−0.07 (−0.17, 0.03)	0.151	2.08 (1.91, 2.24)	2.23 (2.17, 2.29)	−0.15 (−0.31, −0.002)	0.047
Model 2	1.42 (1.30, 1.54)	1.49 (1.42, 1.57)	−0.07 (−0.16, 0.02)	0.117	2.44 (2.30, 2.57)	2.54 (2.49, 2.59)	−0.10 (−0.22, 0.01)	0.086

Model 1 adjusted for age, sex, and race/ethnicity. Model 2 further adjusted for education, marital status, family PIR, physical activity, smoking status, alcohol, total energy, modified HEI, medication for lowering glucose, lipids, and blood pressure (immune domain), and BMI.

## Data Availability

Data are available on the NHANES website, and the analytic codes will be made available pending email request to the corresponding author.

## Supplementary Materials

The following supporting information can be downloaded at: https://www.mdpi.com/article/10.3390/nu16030403/s1, Figure S1: Participant flow chart; Table S1: Structure of AL (domain) score; Table S2: Comparison of the least square means of allostatic load (immune and autonomic) domain scores: total and individual berry consumers versus nonconsumers, *n* = 15,620; Table S3: Comparison of the adjusted least square means of 14 allostatic load biomarkers: berry consumers versus nonconsumers, *n* = 7684. References [49,62,63,64,65,66,67,68,69,70,71,72,73,74,75,103,104,105,106,107,108,109,110,111,112,113,114] are cited in the supplementary materials.
