# Supplementary material for: Berry Consumption in Relation to Allostatic Load in US Adults: The National Health and Nutrition Examination Survey, 2003–2010
Source: Nutrients. 2024 Jan 30;16(3):403. doi: 10.3390/nu16030403 (PMC10857206; doi:10.3390/nu16030403)
Supplement: Supplementary file 1 [file nutrients-16-00403-s001.zip › nutrients-2743412-supplementary.pdf]

Table S1. Structure of AL (domain) score.

Score assignment for each biomarker measurement				
**Measurement**	**Score = 0 (No Risk)**	**Score = 1 (Moderate Risk)**	**Score = 2 (High Risk)**	**References**
**Cardiovascular Domain (0–12)**				
Total cholesterol, mg/dL	<200	200–240	≥240	[75,103]
HDL-cholesterol, mg/dL	>55 (Male)<br>>65 (Female)	40–55 (M)<br>50–65 (F)	<40 (M)<br><50 (F)	[49,64,103–105]
LDL-cholesterol, mg/dL	<100	100–130	≥130	[103,106]
Glucose (fasting), mg/dL	<100	100–125	>125	[65,75]
Insulin (fasting), μU/mL	≥2 or <10	10–25	≥25 or <2	[107,108]
HbA1c, %	<5.7	5.7–6.5	≥6.5	[49,65]
**Autonomic Domain (0–6)**				
Pulse Rate (Beats/Min)	≤60	60–100	≥100	[74,109]
SBP (mm/Hg)	<120	120–140	≥140 mm/Hg or hypertension medication	[62,63,73,75,110]
DBP (mm/Hg)	<80	80–90	≥90 mm/Hg	[62,63,73,75,110]
**Metabolic Domain (0–6)**				
HOMA-IR<br>Defined as fasting glucose × Insulin/22.5, to assess the insulin resistance	<2.6	2.6–4.65	>4.65 or diabetes diagnosis	[66,67,72,111]
Triglycerides (mg/dL)	<150	≥150 and <200	≥200	[68,112]
Waist circumference (cm)	<94 (Male)<br><80 (Female)	94–102 (M)<br>80–88 (F)	>102 (M)<br>>88 (F)	[69]
**Immune Domain (0–4)**				
White Blood Cell Count (1000 cells/μL)	≥1.5 and <4.5	4.5–11	>11 or <1.5	[70,71,113]
CRP (mg/dL)	<0.3	0.3–1	>1	[104,108,114]

Table S2. Comparison of the least square means of allostatic load (immune and autonomic) domain scores: total and individual berry consumers versus nonconsumers, n = 15,620.

Total and Subtype Berries	Immune Domain (white Blood Cells Counts, CRP) (0–4)				Autonomic Domain (pulse Rate, Blood Pressure) (0–6)			
	Consumers (n)	Non-consumers (n)	Difference Estimate	*P* value	Consumers (n)	Non-consumers (n)	Difference Estimate	*P* value
Berries	3026	12594			3026	12594		
Model 1	1.34 (1.30, 1.39)	1.49 (1.46, 1.52)	−0.15 (−0.20, −0.11)	<0.0001	2.17 (2.09, 2.25)	2.34 (2.29, 2.39)	−0.17 (−0.25, −0.09)	<0.0001
Model 2	1.52 (1.45, 1.59)	1.56 (1.51, 1.62)	−0.05 (−0.09, −0.01)	0.029	2.49 (2.40, 2.58)	2.57 (2.49, 2.65)	−0.07 (−0.14, −0.01)	0.0258
Strawberries	1878	13742			1878	13742		
Model 1	1.36 (1.32, 1.41)	1.48 (1.45, 1.51)	−0.12 (−0.16, −0.07)	<0.0001	2.22 (2.13, 2.32)	2.32 (2.27, 2.37)	−0.10 (−0.19, −0.01)	0.038
Model 2	1.548 (1.491, 1.605)	1.552 (1.515, 1.59)	−0.004 (−0.05, 0.04)	0.848	2.55 (2.43, 2.67)	2.63 (2.54, 2.71)	−0.08 (−0.20, 0.04)	0.198
Blueberries	1061	14559			1061	14559		
Model 1	1.31 (1.24, 1.37)	1.48 (1.45, 1.51)	−0.17 (−0.24, −0.10)	<0.0001	2.11 (1.99, 2.23)	2.32 (2.27, 2.37)	−0.21 (−0.32, −0.10)	0.0003
Model 2	1.46 (1.35, 1.57)	1.50 (1.44, 1.56)	−0.04 (−0.14, 0.06)	0.429	2.58 (2.48, 2.68)	2.65 (2.61, 2.69)	−0.07 (−0.17, 0.04)	0.1964
Cranberries	331	15289			331	15289		
Model 1	1.31 (1.19, 1.42)	1.47 (1.44, 1.50)	−0.16 (−0.27, −0.05)	0.0044	2.09 (1.89, 2.30)	2.31 (2.26, 2.36)	−0.22 (−0.41, −0.03)	0.027
Model 2	1.53 (1.41, 1.64)	1.55 (1.52, 1.59)	−0.03 (−0.13, 0.07)	0.566	2.51 (2.32, 2.69)	2.63 (2.58, 2.69)	−0.13 (−0.29, 0.04)	0.121
Raspberries	195	15425			195	15425		
Model 1	1.41 (1.25, 1.53)	1.47 (1.44, 1.50)	−0.06 (−0.20, 0.09)	0.4536	2.15 (1.94, 2.37)	2.31 (2.26, 2.36)	−0.16 (−0.37, 0.06)	0.145
Model 2	1.58 (1.44, 1.73)	1.55 (1.49, 1.61)	0.04 (−0.11, 0.18)	0.613	2.59 (1.98, 2.43)	2.63 (2.57, 2.69)	−0.04 (−0.24, 0.15)	0.669
Blackberries	98	15522			98	15522		
Model 1	1.20 (0.99, 1.41)	1.47 (1.44, 1.50)	−0.27 (−0.49, −0.06)	0.014	1.84 (1.52, 2.16)	2.31 (2.26, 2.36)	−0.47 (−0.79, −0.15)	0.0044
Model 2	1.36 (1.14, 1.59)	1.56 (1.52, 1.60)	−0.20 (−0.42, 0.03)	0.089	2.34 (2.13, 2.56)	2.63 (2.57, 2.69)	−0.29 (−0.51, −0.08)	0.007
Cranberry juice	936	14684			936	14684		
Model 1	1.41 (1.35, 1.47)	1.47 (1.44, 1.50)	−0.07 (−0.12, −0.01)	0.024	2.25 (2.11, 2.39)	2.31 (2.26, 2.36)	−0.06 (−0.20, 0.07)	0.334
Model 2	1.51 (1.43, 1.58)	1.56 (1.52, 1.60)	−0.05 (−0.12, 0.01)	0.105	2.58 (2.46, 2.70)	2.63 (2.57, 2.69)	−0.05 (−0.15, 0.04)	0.277

Model 1 adjusted for age, sex, and race/ethnicity.
Model 2 further adjusted for education, marriage status, family poverty to income ratio, physical activity, smoking status, alcohol consumption, total energy, modified healthy eating index, medication for lowering glucose, lipids, and blood pressure (immune domain), and BMI.

Table S3. Comparison of the adjusted least square means of 14 allostatic load biomarkers: berry consumers versus nonconsumers, n = 7684.

AL Risk Factors	Consumers	Nonconsumers	Model 1		Model 2	
	LSM (95% CI)	LSM (95%CI)	Difference (95% CI)	*p*-Value	Difference (95% CI)	*p*-Value
Pulse (beats/min)						
Berry	68.5 (67.7, 69.3)	71.0 (70.4, 71.5)	−2.5 (−3.3, −1.7)	<0.0001	−1.4 (−2.2, −0.6)	0.0006
Strawberry	69.4 (68.5, 70.3)	70.7 (70.1, 71.2)	−1.3 (−2.1, −0.4)	0.005	−0.6 (−1.6, 0.5)	0.287
Blueberry	68.0 (66.8, 69.3)	70.7 (70.1, 71.2)	−2.6 (−4.0, −1.3)	0.0002	−2.2 (−3.8, −0.6)	0.0067
Cranberry	66.4 (64.4, 68.3)	70.6 (70.1, 71.1)	−4.2 (−6.1, −2.3)	<0.0001	−2.9 (−4.9, −0.9)	0.0054
Raspberry	68.3 (66.3, 70.3)	70.6 (70.0, 71.1)	−2.2 (−4.2, −0.2)	0.0284	−0.9 (−2.9, 1.1)	0.36
Blackberry	65.9 (62.8, 69.1)	70.6 (70.0, 71.1)	−4.6 (−7.8, −1.4)	0.0059	−3.7 (−7.2, −0.21)	0.0382
Cranberry juice	69.4 (68.2, 70.6)	70.6 (70.1, 71.1)	−1.2 (−2.4, −0.02)	0.0471	−1.1 (−2.6, 0.4)	0.1632
SBP (mm Hg)						
Berry	122.4 (121.4,123.4)	124.4 (123.5, 125.3)	−2.0 (−3.1, −1.0)	0.0003	−0.7 (−1.7, 0.31)	0.172
Strawberry	123.1 (121.9,124.2)	124.2 (123.4, 125.1)	−1.2 (−2.2, −0.1)	0.0399	−0.02 (−1.1, 1.1)	0.96
Blueberry	121.8 (119.9,123.7)	124.2 (123.4, 125.0)	−2.4 (−4.4, −0.5)	0.0146	−1.1 (−3.0, 0.7)	0.23
Cranberry	120.2 (117.4,123.1)	124.2 (123.3, 125.0)	−3.9 (−6.8, −1.0)	0.02	−1.9 (−4.6, 0.8)	0.173
Raspberry	120.6 (118.0,123.3)	124.1 (123.3, 124.9)	−3.5 (−6.1, −0.9)	0.0085	−2.1 (−4.9, 0.9)	0.168
Blackberry	119.3 (115.8,122.9)	124.1 (123.3, 124.9)	−4.8 (−8.5, −1.1)	0.0126	−1.7 (−5.8, 2.4)	0.41
Cranberry juice	122.8 (120.7,124.9)	124.2 (123.4, 125.0)	−1.4 (−3.3, 0.55)	0.1574	−1.3 (−3.6, 0.9)	0.226
DBP (mm Hg)						

Berry	69.1 (68.3, 70.0)	69.3 (68.6, 70.0)	−0.2 (−1.1, 0.7)	0.6522	−0.4 (−1.2, 0.5)	0.401
Strawberry	69.3 (68.3, 70.3)	69.3 (68.6, 70.0)	0.01 (−0.94, 0.96)	0.983	−0.2 (−1.1, 0.7)	0.633
Blueberry	68.8 (67.5, 70.0)	69.3 (68.7, 70.0)	−0.6 (−1.9, 0.8)	0.422	−0.6 (−2.0, 0.9)	0.388
Cranberry	69.0 (66.7, 71.2)	69.3 (68.7, 69.9)	−0.4 (−2.5, 1.8)	0.745	−0.2 (−2.3, 1.9)	0.861
Raspberry	71.1 (68.9, 73.2)	69.3 (68.6, 69.9)	1.8 (−0.5, 4.0)	0.118	1.3 (−0.8, 3.4)	0.21
Blackberry	67.7 (64.1, 71.4)	69.3 (68.7, 70.0)	−1.6 (−5.3, 2.1)	0.401	−1.3 (−5.0, 2.4)	0.491
Cranberry juice	67.2 (65.7, 68.6)	69.4 (68.8, 70.1)	−2.3 (−3.7, −0.8)	0.0022	−1.5 (−3.1, 0.1)	0.057
Total cholesterol (mg/dL)						
Berry	195.5 (192.9,198.2)	198.7 (197.1, 200.4)	−3.2 (−6.3, −0.2)	0.04	−2.9 (−6.0, 0.1)	0.058
Strawberry	195.6 (192.6,198.5)	198.5 (196.9, 200.1)	−3.0 (−6.1, 0.2)	0.068	−2.8 (−5.9, 0.3)	0.076
Blueberry	193.4 (188.1,198.7)	198.5 (196.9, 200.0)	−5.1 (−10.7, 0.5)	0.074	−4.1 (−9.7, 1.5)	0.15
Cranberry	202.6 (191.4,213.8)	198.1 (196.6, 199.6)	4.5 (−6.8, 15.8)	0.431	4.7 (−6.3, 15.6)	0.396
Raspberry	199.2 (189.0,209.4)	198.2 (196.7, 199.7)	1.0 (−9.4, 11.4)	0.848	1.7 (−7.8, 11.2)	0.717
Blackberry	201.8 (189.9,213.7)	198.2 (196.7, 199.7)	3.7 (−8.4, 15.7)	0.548	6.5 (−5.9, 18.8)	0.3
Cranberry juice	194.0 (189.8,198.2)	198.5 (197.0, 200.0)	−4.5 (−8.7, −0.2)	0.04	−1.0 (−6.9, 4.9)	0.74
HbA1c (%)						
Berry	5.69 (5.64, 5.75)	5.78 (5.73, 5.83)	−0.08 (−0.13, −0.04)	0.001	−0.03 (−0.07, 0.01)	0.17
Strawberry	5.70 (5.64, 5.75)	5.77 (5.72, 5.82)	−0.07 (−0.12, −0.02)	0.006	−0.03 (−0.08, 0.01)	0.112
Blueberry	5.70 (5.62, 5.78)	5.77 (5.72, 5.82)	−0.07 (−0.14, 0.001)	0.055	−0.03 (−0.09, 0.03)	0.298
Cranberry	5.63 (5.55, 5.71)	5.77 (5.72, 5.81)	−0.14 (−0.21, −0.06)	0.0004	−0.04 (−0.11, 0.04)	0.363
Raspberry	5.69 (5.58, 5.80)	5.76 (5.72, 5.81)	−0.07 (−0.17, 0.03)	0.15	−0.06 (−0.14, 0.03)	0.173
Blackberry	5.72 (5.55, 5.88)	5.76 (5.72, 5.81)	−0.05 (−0.2, 0.11)	0.565	0.005 (−0.12, 0.13)	0.94
Cranberry juice	5.70 (5.62, 5.78)	5.77 (5.72, 5.82)	−0.07 (−0.14, 0.001)	0.0518	−0.04 (−0.13, 0.04)	0.313

HDL-C (mg/dL)						
Berry	56.2 (55.0, 57.4)	53.4 (52.8, 54.1)	2.8 (1.5, 4.1)	<0.0001	1.1 (−0.1, 2.2)	0.063
Strawberry	55.9 (54.5, 57.3)	53.7 (53.1, 54.3)	2.2 (0.8, 3.7)	0.003	0.6 (−0.6, 1.9)	0.338
Blueberry	56.3 (54.5, 58.0)	53.8 (53.2, 54.4)	2.5 (0.7, 4.3)	0.006	0.9 (−0.7, 2.4)	0.264
Cranberry	58.0 (55.1, 60.8)	53.8 (53.3, 54.4)	4.1 (1.4, 6.8)	0.0032	1.0 (−1.3, 3.2)	0.389
Raspberry	55.6 (52.1, 59.1)	53.9 (53.3, 54.5)	1.7 (−1.8, 5.1)	0.3314	0.4 (−2.6, 3.3)	0.802
Blackberry	60.1 (54.1, 66.0)	53.9 (53.3, 54.5)	6.2 (0.3, 12.1)	0.04	2.5 (−3.0, 8.1)	0.362
Cranberry juice	54.2 (52.4, 56.1)	53.9 (53.3, 54.5)	0.3 (−1.5, 2.2)	0.728	−0.4 (−2.5, 1.7)	0.681
LDL-C (mg/dL)						
Berry	114.0 (111.9,116.1)	117.5 (115.9, 119.0)	−3.5 (−5.9, −1.1)	0.006	−2.9 (−5.2, −0.7)	0.012
Strawberry	114.9 (112.3,117.4)	117.1 (115.7, 118.5)	−2.2 (−5.0, 0.5)	0.105	−1.8 (−4.4, 0.8)	0.17
Blueberry	112.3 (108.6,116.1)	117.1 (115.7, 118.5)	−4.8 (−8.7, −0.8)	0.018	−3.7 (−7.5, 0.02)	0.051
Cranberry	118.9 (110.7,127.1)	116.8 (115.5, 118.2)	2.1 (−6.1, 10.3)	0.612	2.7 (−5.2, 10.5)	0.496
Raspberry	118.1 (108.4,127.8)	116.8 (115.5, 118.2)	1.3 (−8.6, 11.2)	0.798	2.3 (−6.8, 11.4)	0.618
Blackberry	121.6 (110.5,132.7)	116.8 (115.5, 118.2)	4.8 (−6.4, 16.0)	0.394	8.0 (−2.9, 19.0)	0.146
Cranberry juice	113.2 (109.4,117.0)	117.1 (115.7, 118.5)	−3.9 (−7.9, −0.02)	0.049	−1.6 (−7.1, 3.8)	0.547
Fasting glucose (mg/dL)						
Berry	106.0 (104.1,108.1)	109.6 (107.9, 111.2)	−3.5 (−5.1, −1.9)	<0.0001	−1.9 (−3.5, −0.3)	0.021
Strawberry	106.2 (103.8,108.5)	109.3 (107.7, 110.9)	−3.1 (−4.9, −1.3)	0.0009	−2.0 (−3.8, −0.2)	0.0299
Blueberry	105.5 (103.4,107.7)	109.2 (107.5, 110.8)	−3.6 (−5.5, −1.8)	0.0003	−2.3 (−4.1, −0.5)	0.011
Cranberry	105.7 (102.8,108.7)	109.0 (107.4, 110.6)	−3.3 (−6.0, −0.5)	0.022	−0.2 (−3.0, 2.6)	0.887
Raspberry	105.1 (101.7,108.6)	109.0 (107.4, 110.6)	−3.9 (−6.9, −0.8)	0.013	−3.7 (−6.4, −0.9)	0.009
Blackberry	104.6 (99.8, 109.4)	109.0 (107.4, 110.6)	−4.4 (−9.0, 0.24)	0.063	−2.5 (−8.4, 3.3)	0.385

Cranberry juice	106.4 (103.8,109.0)	109.1 (107.5, 110.8)	−2.7 (−4.9, −0.5)	0.017	−2.0 (−4.7, 0.7)	0.139
Fasting insulin (μU/mL)						
Berry	11.0 (10.3, 11.8)	12.9 (12.4, 13.4)	−1.9 (−2.6, −1.1)	<0.0001	−0.8 (−1.4, −0.2)	0.009
Strawberry	11.3 (10.5, 12.2)	12.7 (12.3, 13.2)	−1.4 (−2.2, −0.6)	0.0011	−0.4 (−1.1, 0.3)	0.222
Blueberry	10.4 (9.6, 11.2)	12.7 (12.2, 13.2)	−2.3 (−3.1, −1.5)	<0.0001	−1.4 (−2.0, −0.7)	<0.0001
Cranberry	10.2 (9.0, 11.3)	12.6 (12.2, 13.1)	−2.5 (−3.6, −1.4)	<0.0001	−0.5 (−1.4, 0.5)	0.326
Raspberry	12.8 (11.1, 14.5)	12.6 (12.1, 13.0)	0.24 (−1.4, 1.9)	0.771	0.9 (−0.4, 2.1)	0.184
Blackberry	9.8 (7.4, 12.2)	12.6 (12.1, 13.1)	−2.8 (−5.2, −0.4)	0.021	−0.3 (−1.7, 1.0)	0.622
Cranberry juice	11.8 (10.4, 13.1)	12.6 (12.2, 13.1)	−0.9 (−2.1, 0.4)	0.184	−0.7 (−2.0, 0.6)	0.257
Triglycerides (mg/dL)						
Berry	127.4 (121.1,133.6)	142.7 (138.7, 146.8)	−15.4 (−22.4, −8.3)	<0.0001	−7.5 (−14.4, −0.7)	0.0314
Strawberry	126.9 (118.6,135.2)	141.7 (137.8, 145.6)	−14.8 (−23.7, −6.0)	0.0013	−7.9 (−16.4, 0.6)	0.068
Blueberry	121.8 (112.1,131.6)	141.2 (137.4, 145.0)	−19.3 (−29.4, −9.3)	0.0003	−11.1 (−21.0, −1.3)	0.027
Cranberry	125.8 (108.1,143.4)	140.4 (136.6, 144.1)	−14.6 (−32.4, 3.1)	0.105	−0.22 (−16.9, 16.4)	0.979
Raspberry	128.1 (109.3,146.9)	140.2 (136.5, 143.9)	−12.1 (−30.7, 6.4)	0.196	−6.8 (−25.4, 11.7)	0.464
Blackberry	102.0 (83.9, 120.0)	140.3 (136.6, 144.1)	−38.4 (−56.9, −19.8)	0.0001	−20.8 (−38.1, −3.5)	0.019
Cranberry juice	134.8 (124.6,145.0)	140.5 (136.7, 144.2)	−5.6 (−15.6, 4.4)	0.264	4.8 (−6.9, 16.4)	0.42
HOMA-IR						
Berry	3.1 (2.8, 3.4)	3.6 (3.5, 3.8)	−0.6 (−0.8, −0.3)	<0.0001	−0.24 (−0.45, −0.04)	0.0221
Strawberry	3.2 (2.8, 3.5)	3.6 (3.4, 3.8)	−0.4 (−0.7, −0.1)	0.0054	−0.1 (−0.4, 0.1)	0.3533
Blueberry	2.9 (2.6, 3.1)	3.6 (3.4, 3.8)	−0.7 (−1.0, −0.5)	<0.0001	−0.4 (−0.6, −0.3)	<0.0001
Cranberry	2.8 (2.4, 3.1)	3.6 (3.4, 3.7)	−0.8 (−1.1, −0.5)	<0.0001	−0.16 (−0.44, 0.11)	0.247
Raspberry	3.5 (2.9, 4.1)	3.6 (3.4, 3.7)	−0.04 (−0.57, 0.49)	0.872	0.11 (−0.3, 0.5)	0.578
Blackberry	2.7	3.6	−0.9	0.017	−0.18	0.41

	(2.0, 3.4)	(3.4, 3.7)	(−1.5, −0.2)		(−0.6, 0.3)	
Cranberry juice	3.2 (2.8, 3.6)	3.6 (3.4, 3.7)	−0.3 (−0.7, 0.06)	0.095	−0.26 (−0.65, 0.14)	0.1973
WC (cm)						
Berry	95.9 (95.0, 96.9)	99.3 (98.7, 99.9)	−3.4 (−4.3, −2.5)	<0.0001	−1.2 (−1.8, −0.7)	<0.0001
Strawberry	96.1 (94.9, 97.2)	99.1 (98.4, 99.7)	−3.0 (−4.1, −1.8)	<0.0001	−1.1 (−1.7, −0.4)	0.0014
Blueberry	95.4 (93.6, 97.2)	98.9 (98.3, 99.5)	−3.5 (−5.3, −1.7)	0.0002	−1.6 (−2.5, −0.6)	0.0012
Cranberry	93.4 (90.7, 96.0)	98.8 (98.2, 99.4)	−5.4 (−8.0, −2.9)	<0.0001	−1.3 (−2.7, 0.03)	0.055
Raspberry	98.8 (95.3, 102.3)	98.7 (98.1, 99.3)	0.1 (−3.4, 3.5)	0.969	0.39 (−1.7, 2.5)	0.714
Blackberry	93.2 (87.9, 98.6)	98.8 (98.1, 99.4)	−5.5 (−11.0, −0.07)	0.047	−0.68 (−4.0, 2.6)	0.682
Cranberry juice	97.8 (95.8, 99.9)	98.8 (98.2, 99.4)	−0.9 (−2.9, 1.1)	0.355	−0.28 (−1.4, 0.8)	0.614
White Blood Cells (1000 cells/uL)						
Berry	6.2 (6.1, 6.4)	6.8 (6.7, 6.9)	−0.6 (−0.7, −0.4)	<0.0001	−0.3 (−0.4, −0.1)	0.0032
Strawberry	6.3 (6.2, 6.5)	6.8 (6.7, 6.9)	−0.4 (−0.6, −0.2)	<0.0001	−0.1 (−0.3, 0.1)	0.245
Blueberry	6.3 (6.0, 6.6)	6.7 (6.6, 6.9)	−0.4 (−0.8, −0.1)	0.0129	−0.2 (−0.5, 0.1)	0.22
Cranberry	6.3 (5.7, 6.9)	6.7 (6.6, 6.8)	−0.4 (−1.0, 0.2)	0.145	0.03 (−0.5, 0.6)	0.91
Raspberry	6.4 (6.0, 6.9)	6.7 (6.6, 6.8)	−0.3 (−0.7, 0.1)	0.176	0.04 (−0.4, 0.5)	0.86
Blackberry	5.7 (5.0, 6.3)	6.7 (6.6, 6.8)	−1.1 (−1.7, −0.4)	0.0019	−0.5 (−1.0, 0.1)	0.094
Cranberry juice	6.5 (6.3, 6.7)	6.7 (6.6, 6.8)	−0.2 (−0.5, −0.02)	0.0321	−0.3 (−0.5, −0.01)	0.04
CRP (mg/dL)						
Berry	0.39 (0.33, 0.45)	0.44 (0.40, 0.49)	−0.05 (−0.11, 0.004)	0.0668	0.001 (−0.05, 0.05)	0.959
Strawberry	0.40 (0.33, 0.47)	0.44 (0.39, 0.48)	−0.04 (−0.11, 0.03)	0.274	0.01 (−0.05, 0.08)	0.648
Blueberry	0.38 (0.28, 0.47)	0.44 (0.39, 0.48)	−0.06 (−0.15, 0.04)	0.226	−0.01 (−0.1, 0.07)	0.749
Cranberry	0.32 (0.22, 0.43)	0.44 (0.39, 0.48)	−0.11 (−0.21, −0.02)	0.021	−0.02 (−0.1, 0.07)	0.684
Raspberry	0.40 (0.28, 0.52)	0.43 (0.39, 0.48)	−0.03 (−0.14, 0.08)	0.586	0.02 (−0.1, 0.13)	0.776
Blackberry	0.19	0.44	−0.25	<0.0001	−0.15	<0.0001

	(0.11, 0.27)	(0.39, 0.48)	(−0.32, −0.17)		(−0.21, −0.09)	
Cranberry juice	0.39<br>(0.29, 0.50)	0.44<br>(0.39, 0.48)	−0.04<br>(−0.14, 0.05)	0.39	−0.06<br>(−0.18, 0.06)	0.309

Model 1 adjusted for age, sex, and race/ethnicity.

Model 2 further adjusted for education, marriage status, family poverty to income ratio, physical activity, smoking status, alcohol consumption, total energy, modified healthy eating index, medication for lowering glucose, lipids, and blood pressure, and BMI.

LSM: least square mean

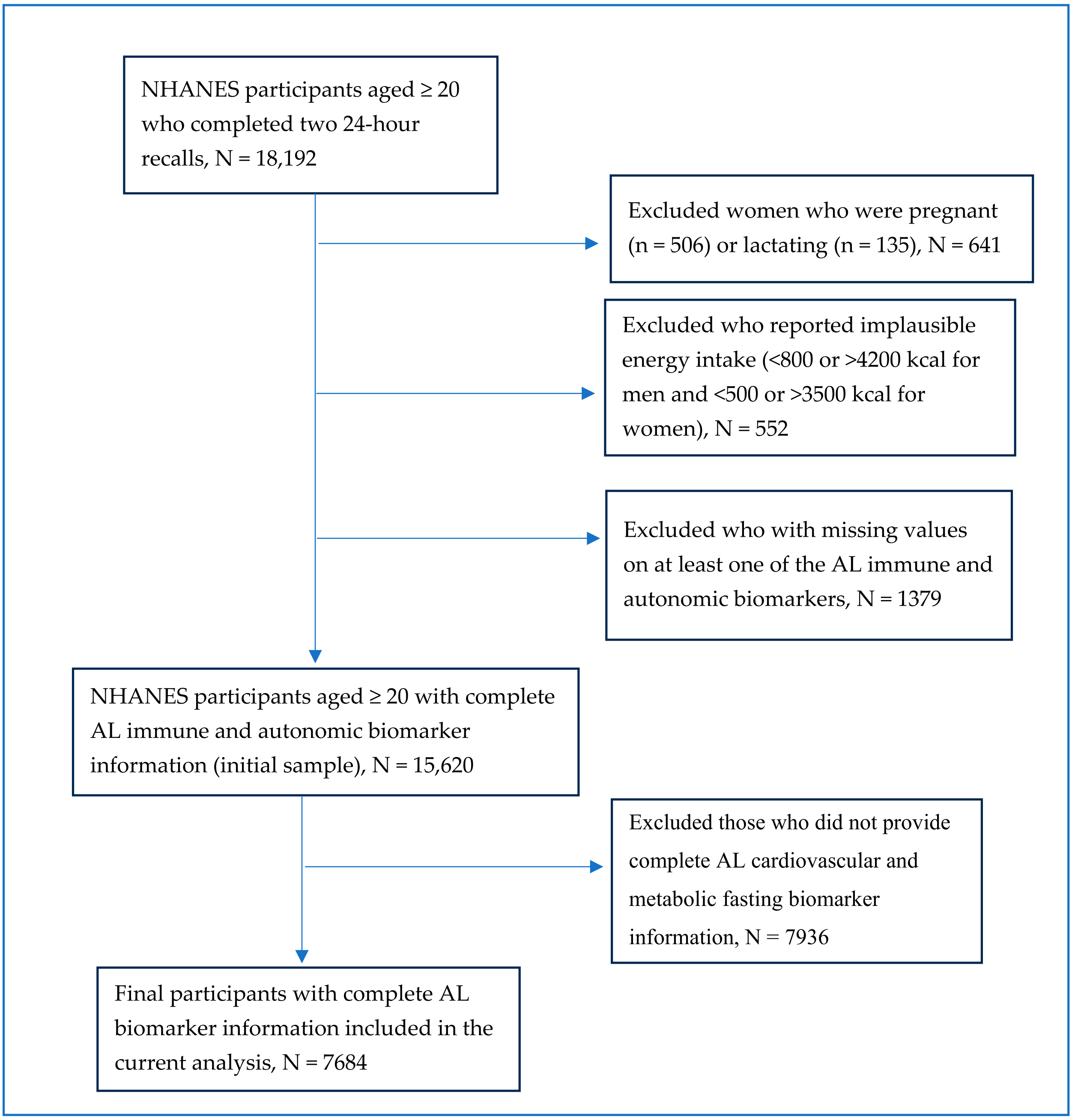

Figure S1. Participant flow chart.
